# Microbial sophorolipids inhibit colorectal tumour cell growth in vitro and restore haematocrit in Apc^min+/−^ mice

**DOI:** 10.1007/s00253-022-12115-6

**Published:** 2022-08-15

**Authors:** Breedge Callaghan, Matthew S. Twigg, Niki Baccile, Inge N. A. Van Bogaert, Roger Marchant, Christopher A. Mitchell, Ibrahim M. Banat

**Affiliations:** 1 School of Biomedical Sciences, Ulster University, Coleraine, BT52 1SA UK; 2 Sorbonne Universités, UPMC Univ Paris 06, CNRS, Collège de France UMR 7574, Chimie de La Matière Condensée de Paris, UMR 7574, 75005 Paris, France; 3 Centre for Synthetic Biology, Department of Biotechnology, Ghent University, Coupure Links 653, 9000 Ghent, Belgium

**Keywords:** Sophorolipids, Acidic, Colorectal, Cancer, Apc^min+/−^

## Abstract

**Abstract:**

Sophorolipids are glycolipid biosurfactants consisting of a carbohydrate sophorose head with a fatty acid tail and exist in either an acidic or lactonic form. Sophorolipids are gaining interest as potential cancer chemotherapeutics due to their inhibitory effects on a range of tumour cell lines. Currently, most anti-cancer studies reporting the effects of sophorolipids have focused on lactonic preparations with the effects of acidic sophorolipids yet to be elucidated. We produced a 94% pure acidic sophorolipid preparation which proved to be non-toxic to normal human colonic and lung cells. In contrast, we observed a dose-dependent reduction in viability of colorectal cancer lines treated with the same preparation. Acidic sophorolipids induced apoptosis and necrosis, reduced migration, and inhibited colony formation in all cancer cell lines tested. Furthermore, oral administration of 50 mg kg^−1^ acidic sophorolipids over 70 days to Apc^min+/−^ mice was well tolerated and resulted in an increased haematocrit, as well as reducing splenic size and red pulp area. Oral feeding did not affect tumour numbers or sizes in this model. This is the first study to show that acidic sophorolipids dose-dependently and specifically reduces colon cancer cell viability in addition to reducing tumour-associated bleeding in the Apc^min+/−^ mouse model.

**Key points:**

• *Acidic sophorolipids are produced by yeast species such as Starmerella bombicola*.

• *Acidic sophorolipids selectively killed colorectal cells with no effect on healthy gut epithelia*.

• *Acidic sophorolipids reduced tumour-associated gut bleed in a colorectal mouse model*.

**Graphical abstract:**

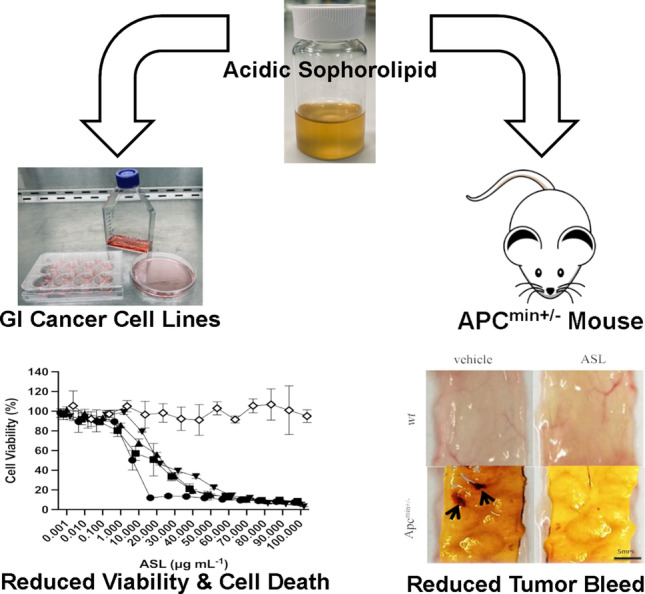

**Supplementary Information:**

The online version contains supplementary material available at 10.1007/s00253-022-12115-6.

## Introduction

Familial adenomatous polyposis (FAP) is an autosomal dominant hereditary form of colorectal cancer which was responsible for over 10% of global cancer incidence and 9.4% of global cancer-related deaths in 2020 (Ferlay et al. 2020). FAP is characterised by the development of numerous adenomas along the colorectal tract. Currently, the gold standard for treatment of FAP is surgery, followed by adjuvant chemotherapy (O’Connell et al. 2010). Adjuvant chemotherapy does not, however, discriminate between normal and transformed tissue, which leads to a variety of potentially serious complications including cardiotoxicity, immune dilapidation, and neurotoxicity (Morgan and Rubin 1998; Mazevet et al. 2013). To allay this problem, compounds that are non-toxic, orally tolerated, and specifically target epithelial neoplastic cells in the intestinal tract could have great potential in delaying progression of intestinal neoplasms; in particular, those that are associated with progression to these colorectal cancers (Kelloff et al. 1994; D’Incalci et al. 2005).

There is an increasing body of research demonstrating compounds derived from natural sources such as plants and microbial secondary metabolites have preventative or targeted anti-cancer activity (Fridlender et al. 2015; Khalifa et al. 2019). An example that is already in widespread in cancer treatment is paclitaxel, first derived from the bark of the yew tree (*Taxus baccata*) (Stierle et al. 1993) and is now used for the treatment of several different cancer types including ovarian, oesophageal, breast, lung, and pancreatic cancer (Barbuti and Chen 2015). Sophorolipids are a class of naturally occurring glycolipids produced by yeast species such as *Starmerella bombicola* (Van Bogaert et al. 2007; Roelants et al. 2019). Comprising a hydrophilic sophorose moiety covalently linked to hydrophobic hydroxylated fatty acid tails ranging between 16 and 18 carbons in length, sophorolipid congeners are produced in two main forms either lactonic (LSL) or acidic (ASL) (Fig. S1) (Banat et al. 2010; Marchant and Banat 2012a, 2012b). During fermentation, yeast species generate mixtures of these structurally different congeners (Van Bogaert et al. 2007). The anti-cancer properties of sophorolipids have received a lot of attention in recent years. Sophorolipids and their synthetically synthesised derivatives have shown in vitro cytotoxic effects in human pancreatic (HPAC), liver (H7402), lung (A549), brain (LN229, HNCG-2), oesophageal (KYSE109, KYSE450), breast, cervivcal (HeLa), leukaemic (HL60, K562), and melanoma (SK-MEL-28) cell lines (Chen et al. 2006a, 2006b; Fu et al. 2008; Dhar et al. 2011; Shao et al. 2012; Ribeiro et al. 2015; Li et al. 2017; Ceresa et al. 2021; Adu et al. 2022). Sophorolipids have also shown potential tumour shrinking capability in an in vivo model of cervical cancer (Li et al. 2017). However, to date*,* very few in vivo bioactivity studies have been reported for sophorolipid mixtures. A small number of toxicology experiments have shown that sophorolipids are non-irritating when topically applied to the skin and eyes of rabbits and non-toxic when administered orally to either mice or rats (Ikeda et al. 1986; Callaghan et al. 2016). Sophorolipid mixtures have also been observed to reduce inflammation and reduce mortality rates in a rat model of severe abdominal sepsis as well as decreasing IgE levels in a murine asthma model (Hardin et al. 2007; Bluth et al. 2008).

Although sophorolipids show promising anti-cancer activity in vitro, the gross composition and percentage of congeners within the sophorolipid mixture used in most investigations are not fully disclosed. It can be assumed that LSL enriched preparations are more common, due to the preponderance of LSLs in comparison to ASLs within naturally occurring sophorolipid mixtures (Van Bogaert et al. 2011; Marchant and Banat 2012a). The composition of sophorolipid preparations is an important factor to consider when investigating the targeted or preventative anti-cancer effect of these molecules. The reasoning for this is twofold: firstly, to reduce toxicity resulting from contaminants in crude preparations and natural products being investigated must be composed completely of highly purified biologically active agent (Beutler 2019; Adu et al. 2022). In the case of pactitaxel and other naturally derived agents, this has necessitated the synthetic production in the laboratory of the active agent with targeted anti-cancer properties (Flam 1994). Secondly, and arguably more important, there is significant difference in the functional properties attributed to LSL and ASL congeners. LSLs have strong antimicrobial action that is not seen in ASLs (Van Bogaert et al. 2007; Elshikh et al. 2017). Previously, we have shown that a highly purified preparation of LSL results in exacerbation of adenomatous tumour growth in the intestinal tract of a colorectal cancer murine model, (Apc^min+/−^ mouse), with secondary consequences including splenomegaly and reduced haematocrit levels (Callaghan et al. 2016).

To date, the anti-cancer bioactivity of purified ASLs has only been investigated within melanoma cell lines (Adu et al. 2022). In contrast to our previous work with LSL, an ASL specific targeted anti-cancer effect would be advantageous for several reasons. In comparison to LSL congeners, ASLs have reduced production costs as they are the first congeners to be produced in the bioreactor when less favourable conditions that require a reduced energy requirement are present (Casas and García-Ochoa 1999; Sarubbo et al. 2022). The proportion of ASL within enriched preparations can be improved when the medium is supplemented with polyunsaturated fatty acids and this can be further increased via simple hydrolysis of the sophorolipid mixtures which increases the number of open ring structures (Van Bogaert et al. 2011). Alternatively, a modified strain lacking the lactonising enzyme can be applied. Yet, in contrast to the hydrolysis strategy, acetylation of the glucose moieties is still possible (Roelants et al. 2016). ASLs have enhanced solubility when compared to other sophorolipid congeners, which may prove useful for their use as pharmaceuticals, as they can be dissolved in saline instead of potentially toxic solvents such as DMSO (Baccile et al. 2016). Both the solubility and enhanced foaming attributes of ASLs contribute to their preferred use in the food, cosmetic, and the bioremediation industries (Roelants et al. 2016; Naughton et al. 2019; Adu et al. 2020).

In this study, we investigated the targeted therapeutic effect of purified ASL in vitro in human colorectal cancer cell lines and their ability to inhibit tumour growth in Apc^min+/−^mice. The Apc^min+/−^ mouse model of FAP was used as it recapitulates key pathological features of the human disease and provides a useful tool to investigate the effects of genetics, diet, and therapeutic drugs on tumorigenesis in the gastrointestinal tract (Hinoi et al. 2007). Like human FAP, it was first noted that Apc^min+/−^ mice develop adult-onset anaemia with haematocrit levels < 45%, the passage of bloody stools, and have a reduced life span (Moser et al. 1995). Additionally, post-mortem analysis revealed the growth of numerous tumours along the small intestine (Moser et al. 1995). The Apc^min+/−^ mouse model has also been widely used to test the effects of chemotherapeutics on tumour growth and development such as NSAIDs (aspirin) (Reuter et al. 2002). In addition, the Apc^min+/−^ mouse model has also been used to develop a further understanding of clinically used chemotherapeutic drugs such as 5-fluorouracil, which is widely used in the treatment of CRC (Tucker et al. 2002). Therefore, we hypothesise that a highly purified and well characterised preparation of ASL (94% pure diacetylated) will selectively inhibit colorectal tumour growth in vitro and delay disease progression in the Apc^min+/−^ mouse model.

## Material and methods

### ASL production and purification

ASLs used in this study were generated in house from a crude precursor sophorolipid mixture, (*Sopholiance S*, Batch N°11103A), purchased from *Givaudan* (Vernier, Switzerland). ASL congeners were obtained according to a method described by Baccile et al. (2013). In brief, sophorolipids were purified from *Sopholiance S* via liquid phase extraction in acidified ethyl acetate followed by hexane washing. Following purification, the sophorolipid material was subjected to alkaline hydrolysis by diluting in 5 M NaOH (*Merck*) and heating under reflux to 90 °C for 10 min. The material was then acidified to pH 4 using 18.5% (w/v) HCl (*Merck*). ASL was then recovered via precipitation in a pentanol/hexane medium (*Merck*) at − 18 °C (Baccile et al. 2013). Following production and purification, SL congeners were identified by a UHPLC system with RS Diode Array detector (*ThermoFisher* *Scientific*) in conjunction with the amaZon SL dual funnel Ion Trap spectrometer LCMS system (*Bruker*). The percentage relative amounts of each congener were calculated and a ratio of ASL to LSL determined (Smyth et al. 2010).

### Cell culture

Colorectal cancer cell lines HT29 (ATCC® HTB-38), HT115 (ECACC 85061104), HCT116 (ATCC® CCL-247), and Caco2 (ATCC® HTB-37) as well as colonic epithelium CCD-841-CoN (ATCC® CRL-1790) cell lines were used in this study. Cell lines were maintained in DMEM low glucose media or MEM low glucose media (*ThermoFisher* *Scientific*) supplemented with 10% (v/v) foetal bovine serum (*ThermoFisher* *Scientific*). All cultures were maintained at 37 °C in a humidified atmosphere containing 5% CO_2_.

### Cell viability assay

A total of 1 × 10^4^ cells per well were seeded in to a 96 well plate (*Sarstedt*) and allowed to attach overnight before being serum starved for 24 h. Cells were then treated with concentrations of ASL ranging between 0.001 and 100 µg mL^−1^ or vehicle-only control (PBS) for 24 h. Subsequently, 10 µL of a 25 mg mL^−1^ solution of MTT (3-(4, 5-dimethylthiazol-2-yl) -2, 5-diphenyltetrazolium bromide) (*Merck*) was added to each well and the plate further incubated for 1 h at 37 °C. Formazan crystals were solubilised with 100 µL of DMSO (*Merck*). Absorbance at 570 nm was measured using a FLUOstar Omega microplate reader (*BMG-LABTECH*). Experiments were repeated three times with six internal replicates per treatment group. Data are representative of the three independent repeats and presented as mean percentage reduction in absorbance in comparison to vehicle-only controls ± standard deviation (SD).

### Visualisation of morphological changes induced by ASL treatment

A total of 1 × 10^4^ cells per well were seeded on to a 96 well plate (*Sarstedt*) and allowed to attach overnight forming a monolayer before being serum starved for 24 h. Cells were then treated with both 20 and 70 μg mL^−1^ of ASL or vehicle-only control (PBS) for 24 h. The cells were subsequently imaged with an Axio Scope 1 microscope (*Zeiss*) at 200 × magnification. Images obtained from three random fields were selected and assessed for morphological changes by comparing each cell line to the vehicle-only control, looking for changes in cell shape and confluency. Experiments were plated in triplicate and repeated three times.

### Quantification of detached cells

A total of 1 × 10^4^ cells per well were seeded on to a 96 well plate (*Sarstedt*) and allowed to attach overnight forming a monolayer before being serum starved for 24 h. Cells were then treated with both 40 and 70 μg mL^−1^ of ASL or vehicle-only control (PBS) for 24 h. Ten micrograms of supernatant was removed from each well, placed into a 1.5 mL microtube (*Sarstedt*). Cells were stained with 5 μM of both Syto 9 and propidium iodide (*Merck*) by adding directly into the microtube and incubating at room temperature for 30 min. The cells were then centrifuged at 150 × *g* for 5 min and the supernatant was aspirated. The remaining cells pellet was washed using ice-cold PBS (pH 7.4) (*ThermoFisher* *Scientific*) and spun onto a microscope slide using a Shandon cytocentrifuge (*ThermoFisher* *Scientific*) for 5 min at 176 × *g*. Slides were subsequently imaged with a Axio Scope 1 microscope (*Zeiss*) at 400 × magnification. Following staining with Syto9 and propidium iodide, fluorescent staining was present in nucleus therefore live cells appeared green while dead cells fluoresced red (Altman et al. 1993) and as such cells were counted. A minimum of 1 cell and a maximum of 250 cells were counted per field of view. Experiments were plated in triplicate and repeated three times, data set shown as mean ± standard error of the mean (SEM).

### Acridine orange/ethidium bromide staining and quantification

To determine the mechanism of cell death induced by addition of ASL, cells were stained in situ with acridine orange at 10 mg mL^−1^ (*Merck*) and ethidium bromide 1 mg mL^−1^ (*Merck*). Morphological changes were assessed by fluorescence microscopy. For assessment of apoptosis, a total of 3 × 10^4^ cells were seeded onto a 10 mm coverslip (*Agar Scientific*) placed within a 6 well plate and the cells were incubated overnight to form a confluent monolayer. Following serum starvation for 24 h, ASL (at a concentration of either 20 µg mL^−1^ or 70 µg mL^−1^), vehicle control (PBS), or 5 µM of etoposide (apoptotic control) (*Merck*) was added and the plate incubated for a further 24 h. To determine the number of live cells remaining on the coverslip, the samples were washed three times with ice-cold phosphate buffered saline at pH7.4 (*ThermoFisher* *Scientific*), followed by incubation with 10 µL of a solution of acridine orange/ethidium bromide with a volumetric ratio of 1:1 for 5 min. Finally, the cells were washed again three times with ice-cold PBS at pH7.4 (*ThermoFisher* *Scientific*). Cells were subsequently imaged with a Axio Scope 1 florescence microscope (*Zeiss*) at 400 × magnification. A total of 300 attached cells/coverslip were morphologically identified and counted as necrotic (red/orange nuclei), apoptotic (green condensed or fragmented nuclei), or live (green non-condensed ovoid or rounded nuclei). The operator was blinded to the experimental groups and random fields were selected. Each experiment was replicated three times with six internal repeats per group. Data are representative of three independent repeats and presented as mean ± SEM.

### Scratch assay

For wound healing scratch assays, 1.6 × 10^6^ cells were plated in each well of a 6 well plate (*Sarstedt*) and allowed to attach overnight. Cells were serum starved for 24 h and treated with 5 µg mL^−1^ of mitomycin C (*Merck*) for 2 h prior to the scratch to inhibit proliferation. The “wound” was made by scratching a line in the centre of the confluent monolayer using a sterile toothpick. Cells were rinsed very gently three times with PBS at pH7.4 (*ThermoFisher* *Scientific*) and cultivated in serum free media supplemented with PBS vehicle-control or 10–20 µg mL^−1^ ASL for up to 72 h. The cell was imaged at various time points using an ELWD TI SCP microscope (*Nikon* *Europe B. V.*) at 100 × magnification. To quantify migration of cells into the scratch wound, the area of the gap was measured using ImageJ software (Schneider et al. 2012). After 72 h, the area of the remaining gap was measured and the difference between initial and final areas was calculated. Experiments were repeated three times using triplicate technical replicates per experimental group. Data are representative of the three independent repeats and presented as mean ± SEM.

### Chemotactic cell migration assays

Chemotactic cell migration assays were carried out in a modified Boyden chamber, based on a previously described protocol (Chen 2005). 1 × 10^4^ cells per well of CCD-841-CoN, HT29, or HT115 CRC cells were placed in the upper compartment of a 96-well FluoroBlok transwell inserts (*Analab*) which contained 8 μm pore size polyethylene terephthalate filters (*Analab*) and the cells were allowed to attach overnight. A final concentration of 0, 10, or 50 μg mL^−1^ of ASL in serum free media was added to the upper chamber, while appropriate culture media supplemented with 10% FBS was added (as a chemoattractant) to the bottom chamber. Cells were left to migrate overnight in an incubator at 37 °C and at 5% CO_2_. Following incubation, all media in the upper chamber was removed and all cells that failed to migrate were removed using a sterile cotton swab. Cells that had migrated through to the lower surface of the filter insert were stained with a 0.1% (w/v) solution of crystal violet (*Merck*) made up in 25% (v/v) methanol (*Merck*). A total of three random areas were chosen and a minimum of 1 cell and a maximum of 300 cells stained with crystal violet were counted under a Axio Scope 1 light microscope (*Zeiss*) at 400 × magnification. Migration rates were expressed as total percentage of the control. Data are representative of the three independent repeats and presented as mean ± SEM.

### Animal model

Prior to the commencement of the study, all animal procedures were approved by both the animal care and ethics committee at Ulster University and the UK Home Office. Additionally, all animal procedures were carried out by licensed personnel in accordance with both local animal welfare committee (Ulster University) and national (UK Home Office) guidelines (Workman et al. 2010). For breeding purposes, male Apc^min+/−^ mice were housed together with female wild type (*wt*) mice and all the animals subjected to a 12/12 light cycle, with food and water being available ad libitum. Mice were genotyped as described previously in Callaghan et al. (2016), with both heterozygous Apc^min+/−^ and *wt* mice (male and female) used in experiments. During the study, mice were monitored daily for grooming activity, general behaviour, activity levels, food and water intake, and general wellbeing. Mice assessed as exhibiting signs of distress or discomfort were immediately removed from the study and euthanized via CO_2_ asphyxiation.

### ASL dosing

At 5 weeks of age, both *wt* littermate and Apc^min+/−^ mice were treated via oral gavage every other day with either vehicle-only or a solution containing 50 mg kg^−1^ (body weight) of ASL suspended in saline for 70 days. During treatment, body weights, general health, and behaviour were monitored bi-weekly. Food and water were weighed on a weekly basis to determine the effect of ASL treatment on eating/drinking habits. A cut-off point for body weight reduction of 10% was applied and mice reaching this point were euthanized via CO_2_ euthanasia.

### Tissue collection and assessment

At the end of the experimental protocol, mice were euthanized with an overdose of pentobarbitone (200 mg kg^−1^ given IP). A cardiac puncture was performed to collect blood that was stored in EDTA tubes (*Aquilant Scientific*). An additional blood sample was taken for haematocrit level estimation. For this, a blood capillary tube was filled with blood, sealed, and centrifuged (Microcentrifuge with Haematocrit Rotor; *Cole-Parmer*) at 13,000 × *g* for 5 min. Haematocrit levels were calculated using the following calculation: height of RBC/total height of all the components × 100. Internal organs including the intestinal tract, colon, spleen, heart, liver, kidneys, and lungs were removed, weighed, and immerse fixed in 10% buffered formal saline at pH7.4 (*ThermoFisher* *Scientific*). The intestinal tracts were divided into 3 sections according to the description provided by Casteleyn et al. (2010). After identification of the specific intestinal regions, samples were bisected longitudinally, and the total number of polyps was recorded as well as their diameters measured with callipers (Casteleyn et al. 2010). The specimens were then cut into ~ 2 cm strips and placed in cassettes prior to standard wax embedding. To assess qualitative histopathological changes in the intestines and spleen, tissues were cut into 5 µm sections using a Shandon Finesse 325 Microtome (*ThermoFisher* *Scientific*) placed on glass slides, cleared with xylene, dehydrated in descending grades of ethanol, and subsequently stained with Mayer’s haematoxylin and eosin (*Merck*). Stained sections were examined with a Axio Scope 1 light microscope (*Zeiss*) at a range of objective magnifications.

### Statistical analyses

Statistical analysis of cell viability data was determined by two-way ANOVA followed by both Tukey’s and Dunnett’s post hoc testing. Analysis of live/dead cell counts was determined by two-way ANOVA followed by Šidák’s post hoc testing. Analysis of remaining in vitro data was determined by two-way ANOVA followed by both Dunnett’s post hoc testing. Comparisons between in vivo groups were assessed using a Student’s *t*-test. A value of *p* ≤ 0.05 was considered statistically significant. All statistical analysis was carried out with the aid of Prism Version 9.3.1 (350) (*GraphPad* *Software*).

## Results

### Production and purification of ASL

A purified form of diacetylated ASL was produced for use in this study from a commercially available SL formulation using methodologies outlined by Baccile et al. (2013). Following the recommendation of Twigg et al. (2021), the purified sample was analysed via HPLC-ELSD and was found to be composed of 94% nonacetylated ASL. A breakdown of the various nonacetylated ASL congeners present in the samples can be seen in Table S1. The most abundant congener present in the sample was acidic C18:1 s (75% relative abundance). The acidic C18:1t and C18:2t congeners represented 10% each, while all other ASL congeners were limited to less than 2% relative abundance (Twigg et al. 2021).

### ASL have a selective effect on colorectal cancer cell viability

**Fig. 1:**
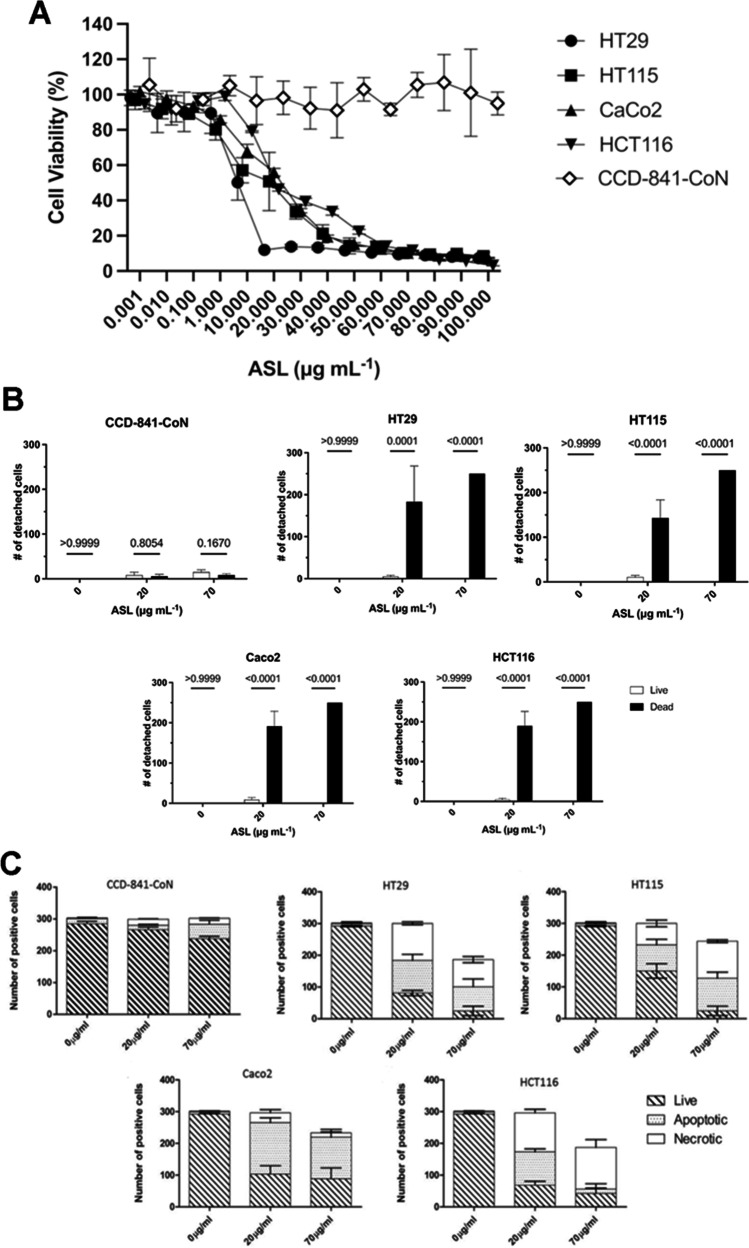
Treatment with ASL results in a detrimental effect on colorectal cancer cell lines in vitro. Compared to normal colonic epithelial cells, the viability of four colorectal cancer cell lines was significantly reduced when treated with ASL (**a**). Treatment with ASL also resulted in increased cell detachment in colorectal cancer cell lines compared to normal colonic epithelial cells (**b**). Colorectal cancer cell lines treated with either 20 or 70 μg mL^−1^ ASL showed indications of both apoptosis and necrosis driven cell death (**c**)

Several colorectal tumour cell lines (HT29, HT115, HCT116, and Caco2), in addition to a colonic epithelial call line (CCD-841-CoN) were treated with ASL to assess the effect on cell viability. ASL at concentrations up to 100 µg mL^−1^ effected no significant decrease in the viability of the CCD-841-CoN cell line after 24 h of treatment in comparison to cultures treated with a vehicle-only control (PBS) (Fig. 1a). However, 24 h of treatment with ASL concentrations equal to or above 1 µg mL^−1^ resulted in significantly reduced viability in the HT115 cell line in comparison to vehicle-only controls (*p* = 0.0201) (Fig. 1a). In HT29, Caco2, and HCT116 cell lines, treatment with ASL concentrations equal to or above 10 µg mL^−1^ resulted in a significant reduction in cell viability in comparison with cells treated with vehicle-only controls (*p* < 0.0001, < 0.0001, and 0.0099 respectively) (Fig. 1a). The viability of colorectal tumour cell lines HT29, HT115, and Caco2 were significantly reduced when compared to the colonic epithelial cell line CCD-841-CoN when treated with ASL concentrations equal to or above 1 µg mL^−1^ (*p* = 0.0146, < 0.0001, and 0.0017 respectively). For cell line HCT116, a significant reduction in viability when compared to CCD-841-CoN was observed when cells were treated with ASL concentrations equal to or above 10 µg mL^−1^ (*p* = 0.0046) (Fig. 1a).

### ASL induces cell rounding, detachment, and cell death in colorectal cancer cells

To observe potential effects on cell morphology, colorectal cancer cell lines (HT29, HT115, HCT116, and Caco2) and the colonic epithelial cell line (CCD-841-CoN) were treated with ASL and assessed by microscopic examination. CCD-841-CoN cells treated with a vehicle-only control (PBS) grew as a confluent monolayer with a bipolar morphology (Fig. S2). Following treatment with either 20 or 70 µg mL^−1^ ASL, no morphological differences of cells in the monolayer were observed when compared to vehicle-only treated CCD-841-CoN cells (Fig. S2). HT29, HT115, HCT116, and Caco2 treated with the vehicle-only control displayed a densely packed, cobblestone-like monolayer morphology (Fig. S2). When treated with ASL, disruption to the monolayer and detachment of HT-29, HT115, HCT116, and Caco-2 cell lines was observed. Partially detached and rounded cells were conspicuous within HT29, HT115, and Caco2 cell lines treated with 20 µg mL^−1^ (Fig. S2). HT29, HT115, HCT116, and Caco2 cells exposed to 70 µg mL^−1^ ASL demonstrated extensive monolayer disruption with distinctive cell free areas within the cultures (Fig. S2).

As cellular detachment was observed in all tumour cell cultures, cells were isolated from the supernatant and stained with syto9 and propidium iodine to determine if detachment resulted in cell death. Only a small number of CD-841-CoN cells treated with either concentration of ASL were observed to detach and there was no significant difference in the proportion of these detached cells that were either alive or dead (Fig. 1b). In contrast, the number of detached HT29, HT115, HCT116, and Caco2 cells was higher when treated with either concentration of ASL when compared to vehicle-only treated cells, and there was a significant increase in the proportion of these detached cells that were found to be dead (Fig. 1b).

### ASL induces cell death in vitro via both apocopic and necrotic pathways

As treatment with ASL resulted in a significant loss of cell viability and induced detachment and cell death in colorectal cancer cell lines, the mechanism of cell death was investigated. Colorectal cancer (HT29, HT115, and HCT116 and Caco2) and colonic epithelial (CCD-841-CoN) cells lines were treated with ASL and stained with acridine orange and ethidium bromide to determine whether cell death was morphologically identifiable as a result of apoptosis or necrosis. Treatment of CCD-841-CoN cells with 20 µg mL^−1^ of ASL did not result in a significant increase in the numbers of either apoptotic or necrotic cells. However, treatment with 70 µg mL^−1^ ASL resulted in 10% cell death within the culture that was identified morphologically as apoptosis (*p* < 0.05) (Fig. 1c). A dose-dependent increase in cell death in all four human colorectal cancer cell lines was observed following ASL treatment (Fig. 1C). In HT29, HT115, and HCT116 cell lines treated with 20 µg mL^−1^ ASL, there were equivalent numbers of apoptotic and necrotic cells. However, there was a higher number of apoptotic cells in Caco2 cells treated with 20 µg mL^−1^ ASL (*p* < 0.01) (Fig. 1c). Exposure of cultures to 70 µg mL^−1^ ASL resulted in a markedly reduced number of adherent colorectal cancer cells available for quantification. HT29 and HT115 cultures treated with 70 µg mL^−1^ showed a significant increase in the numbers of apoptotic and necrotic cells (*p* < 0.001, *p* < 0.01 respectively) while HCT116 cells were predominantly necrotic (*p* < 0.0001), and Caco-2 cells had a higher number of apoptotic cells (*p* < 0.001) compared to vehicle-only controls (Fig. 1c).

### ASL reduces motility and anchorage-independent growth of tumour cells

**Fig. 2:**
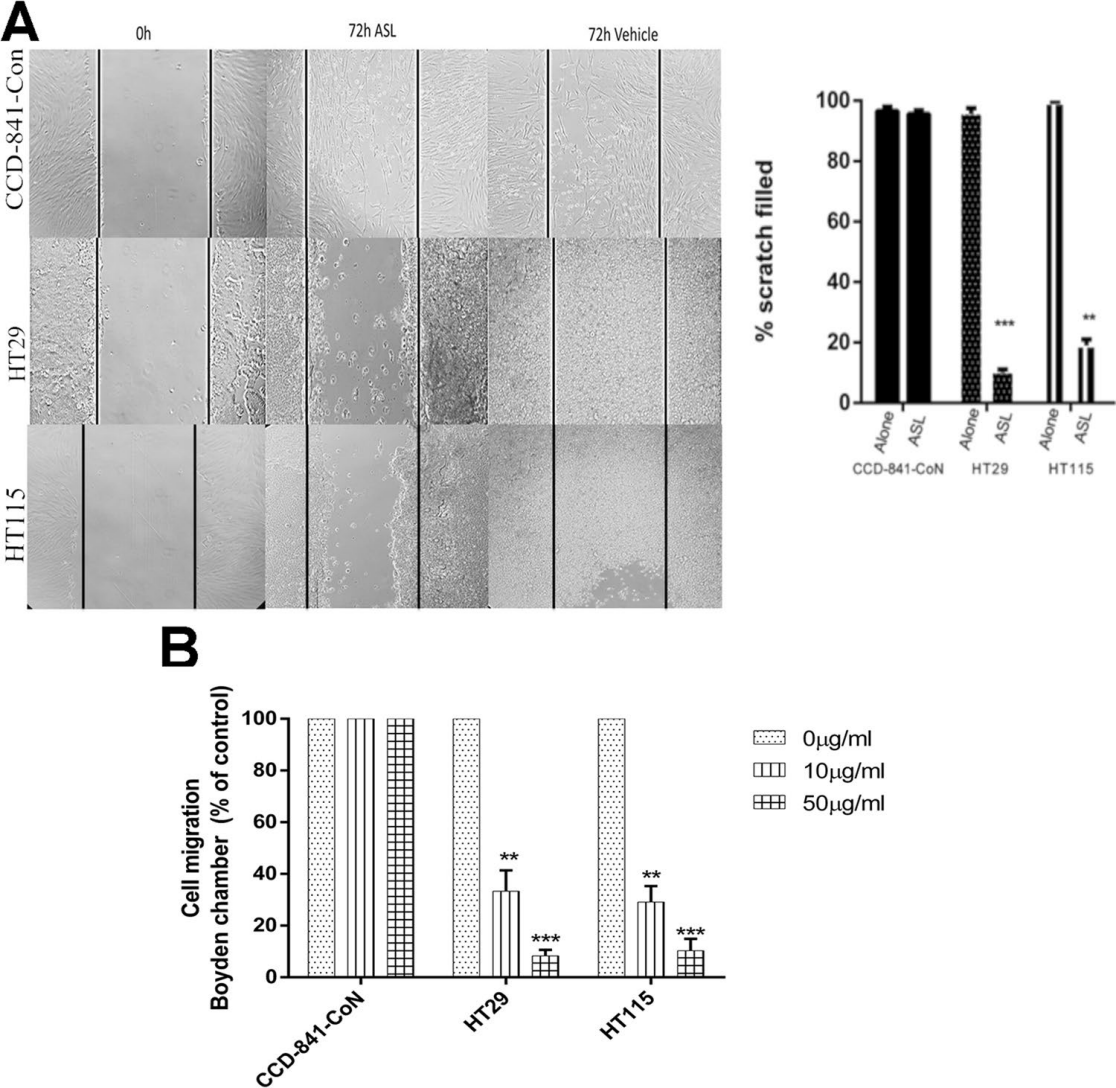
Treatment with ASL significantly reduces cell migration in colorectal cancer cell lines. In a scratch assay ASL, treated colorectal cancer cells showed significantly less migration 72 h post treatment than these treated with vehicle-only controls (**a**). Cell migration across a Boyden chamber in response to a stimulus was significantly reduced in colorectal cancer cells treated with ASL in comparison vehicle-only control treated cells (**b**). Two-way ANOVA (**p < 0.01 and ***p < 0.001)

To assess the effect of ASL on cell migration, a scratch was made across a monolayer of colorectal cell lines HT29 and HCT115, and the colonic epithelial cell line CCD-841-CoN. Treatment with 10 µg mL^−1^ ASL had no significant effect on CCD-841-CoN cell migration, with 90% of the total scratch area being covered after 72 h (Fig. 2a). In contrast, treatment with 10 µg mL^−1^ ASL resulted in a highly significant decrease in the proportion of the total scratch area covered in HT-29 (10%; *p* < 0.0001) and HT115 cells (22%; *p* < 0.0001) after 72 h (Fig. 2a). To test the effect ASL had on chemotaxis, the same cell lines were plated in the upper portion of a Boyden chamber and the number of cells migrating in response to FCS was counted after 24 h. Media supplemented with either 10 or 50 µg mL^−1^ ASL did not significantly affect the migration of CCD-841-CoN cells. The addition of 10 µg mL^−1^ ASL to media did, however, result in a significant reduction in migration of HT29 (33%) and HT115 (29%) compared to control values (*p* < 0.001) (Fig. 2b). The addition of 50 µg mL^−1^ ASL reduced migration of HT29 and HT115 to 8.3% and 10.4% of control values respectively (*p* < 0.0001) (Fig. 2b).

### In vivo experiments: wt and Apc^min+/−^ mice tolerate the oral administration of ASL

To determine palatability and potential toxicity of ASL, a pilot study was performed with both *wt* and Apc^min+/−^ mice which were fed either a vehicle-only control solution consisting of 10% sucrose water or vehicle containing either 0.5, 5, or 50 mg kg^−1^ ASL for a period of 5 weeks (*n* = 3/group). Both *wt* and Apc^min+/−^ mice gained weight at a similar rate and there was no change in food or water intake between any of the treatment groups (Fig. S3). On completion of this oral feeding tolerance study, the major organs (liver, stomach, kidneys, lungs, heart, spleen, and pancreas) were removed and weighed and a gross inspection of morphology was carried out. There were no significant differences in organ weights (Table S2) or gross organ morphology observed between mice fed on the vehicle only control diet or on ASL.

### ASL reduces polyp-associated bleeding and increases haematocrit levels but does not affect tumour size or numbers in Apc^min+/−^ mice

**Fig. 3:**
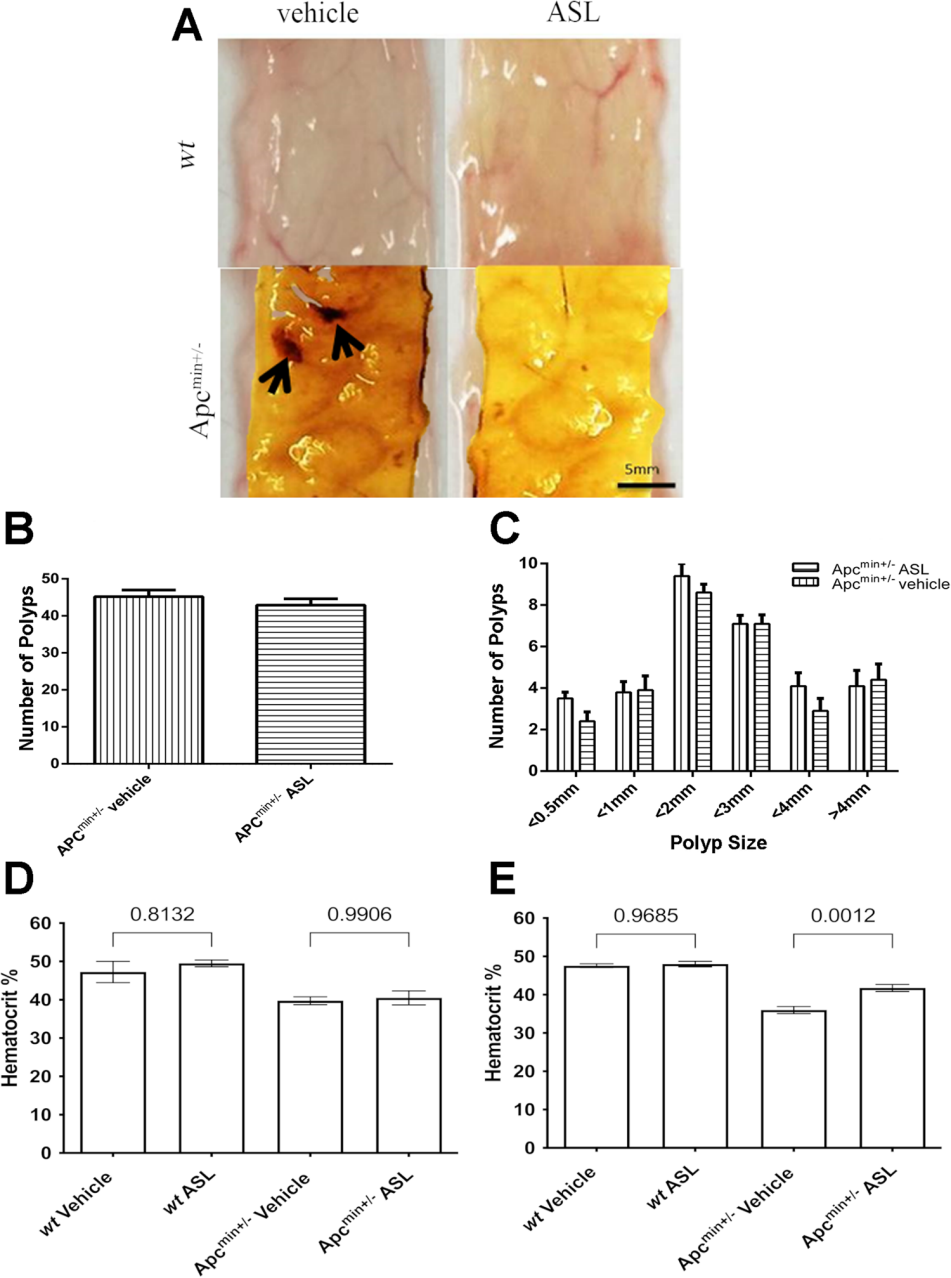
An ASL supplemented diet reduced intestinal bleed in a mouse mode of colorectal cancer. No morphological differences were observed in ileal sections of either wt or Apc^min+/−^ mice fed with either vehicle-only controls or ASL; however, vehicle-only control fed Apc^min+/−^ mice showed evidence of intestinal bleeding from polyps (arrows), whereas ASL fed Apc^min+/−^ mice showed no evidence of intestinal bleeding (**a**). No significant difference in intestinal polyp number (**b**) or polyp sizes (**c**) was observed in Apc^min+/−^ mice fed on either ASL or vehicle-only control. Following 35 days, no significant difference was observed in haematocrit in Apc^min+/−^ mice fed with ASL or vehicle-only control (**d**); however, after 70 days, a significant increase in haematocrit in Apc^min+/−^ mice fed with ASL was observed compared to those fed with vehicle-only control (**e**). One-way ANOVA, *p* values displayed on graphs

The gross morphological appearances of unfixed, flat-mounted ilea from *wt* mice treated with either vehicle-only control or 50 mg kg^−1^ ASL were characterised by a flattened, uniformly smooth mucous epithelium with prominent patent blood vessels (Fig. 3a). In vehicle-only treated Apc^min+/−^ mice, there was clear evidence of polyp-associated bleeding within the ileal segment (Fig. 3a). In contrast, Apc^min+/−^ mice treated with 50 mg kg^−1^ ASL for 70 days showed little evidence of bleeding from these intestinal polyps (Fig. 3a). However, the number of intestinal polyps in Apc^min+/−^ mice was not significantly different following treatment with 50 mg kg^−1^ ASL for 70 days in comparison the vehicle-only control treated cohort (vehicle = 48 ± 2 vs ASL = 45 ± 4; *p* < 0.1) (Fig. 3b). ASL treatments also had no effect on the modal size distribution of the polyps in comparison to vehicle-only treatment (vehicle- 4 mm vs ASL 4 mm; *p* > 0.05) (Fig. 3c).

No significant difference in haematocrit level was observed in *wt* mice fed with either vehicle-only control or 50 mg kg^−1^ ASL for 35 or 70 days (*p* = 0.8132 and *p* = 0.9685 respectively) (Fig. 3d and e). Haematocrit levels in *wt* mice were also significantly higher than those of Apc^min+/−^ mice, irrespective of treatment with either vehicle-only control or ASL (*p* < 0.05) (Fig. 3d and e). After 35 days feeding with 50 mg kg^−1^ ASL, no significant differences in haematocrit levels were observed in Apc^min+/−^ mice compared to those fed with vehicle-only control (vehicle-only = 41.2 ± 0.8 vs ASL = 40.0 ± 1.1; *p* = 0.9906) (Fig. 3d). However, following 70 days of administration with 50 mg kg^−1^ASL, haematocrit levels were found to be significantly higher in Apc^min+/−^ mice fed in comparison to those fed with the vehicle-only control (vehicle-only = 36.0 ± 0.9 vs ASL = 42 ± 1.9; *p* = 0.012) (Fig. 3e).

### ASL effects the splenic weight, proportion of spleen red pulp in Apc^min+/−^ mice

**Fig. 4:**
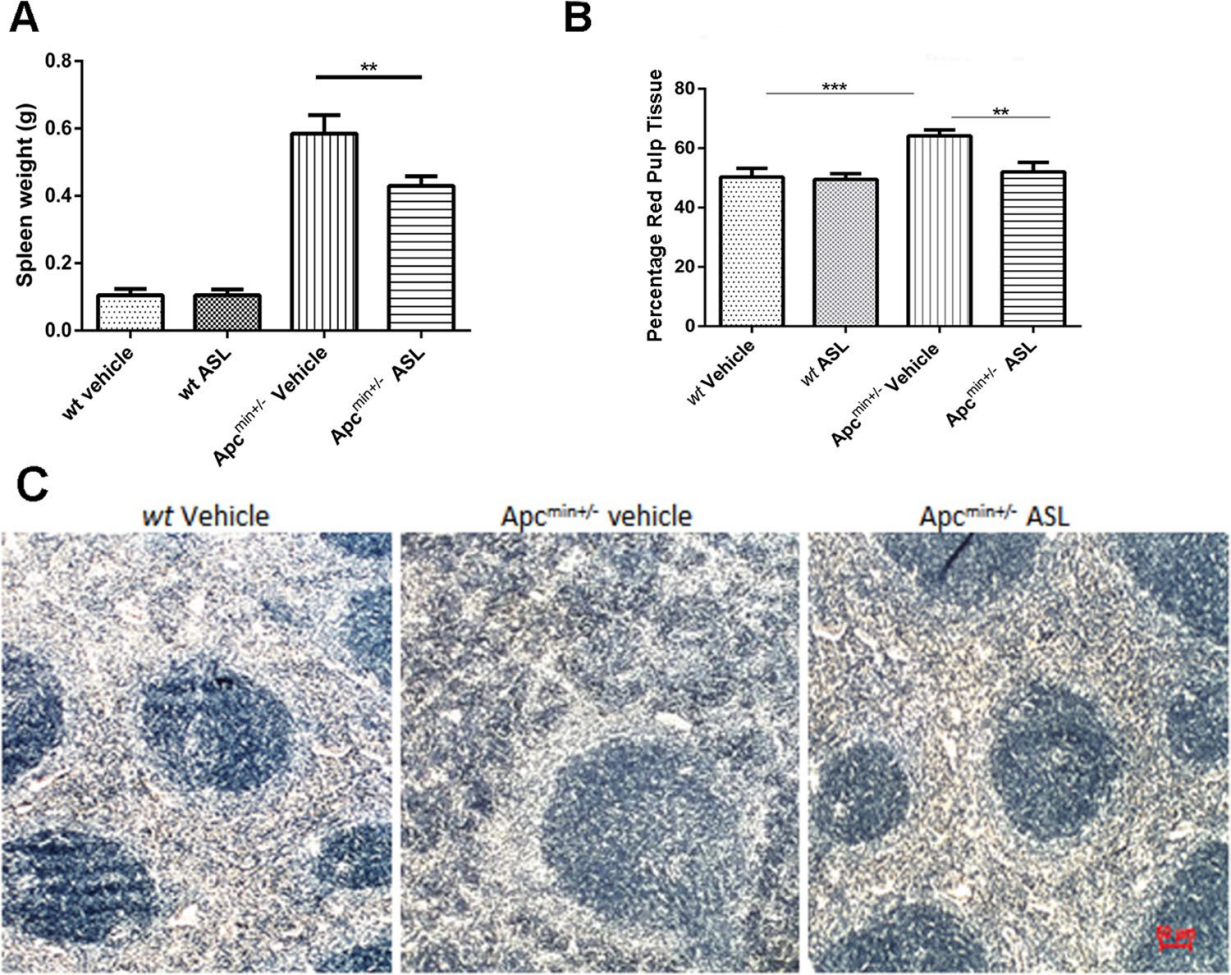
Following 70 days, both splenic weight (**a**) and percentage of red pulp regions (**b**) in Apc^min+/−^ mice fed with ASL were significantly reduced in comparison to those fed with vehicle-only control (*p* < 0.001 and *p* < 0.05 respectively). Apc^min+/−^ mice fed with vehicle-only control had increased red pulp with a loss reticular structure compared to wild-type mice. Splenic morphology (particularly in white pulp) after feeding Apc^min+/−^ mice with ASL more closely resembled that seen in wild-type mice (**c**)

The spleens from vehicle-only control fed Apc^min+/−^ mice were significantly heavier than their *wt* littermates (0.58 ± 0.2 g vs 0.15 g ± 0.5 g; *p* < 0.0001). A dose of 50 mg kg^−1^ ASL had no effect on splenic weight in *wt* mice after 70 days of treatment. However, administration of 50 mg kg^−1^ ASL for 70 days to Apc^min+/−^ mice resulted in a statistically significant decrease in splenic weight (0.58 ± 0.2 g vs 0.40 ± 0.4 g; *p* < 0.001) (Fig. 4a). Following treatment of the Apc^min+/−^ mice with 50 mg kg^−1^ ASL, there was a significant reduction in the proportion of red pulp as compared with vehicle-only controls (62 ± 3.2 vs 48 ± 1.4; *p* < 0.001), and no significant difference was observed in the *wt* mice (Fig. 4b). Histological examination of sections from *wt* mouse spleen revealed conspicuous and intensely basophilic areas of white pulp; these were separated by less dense regions of red pulp; areas that are responsible for removal of old or damaged erythrocytes. In vehicle-only control treated Apc^min+/−^ mice, there was obvious clumping of the red pulp, and the proportion of this tissue was significantly increased when compared to *wt* mice (Fig. 4c). Apc^min+/−^ mice fed with ASL showed reduced clumping of red pulp and an increase in the white pulp regions, like that of the *wt* mice (Fig. 4c).

## Discussion

The ability of chemotherapeutic agents to selectively target cancerous cells while sparing normal tissue is a highly desirable trait, as it may mitigate against the common side effects associated with these toxic therapeutics such as epithelial cell damage in the gastrointestinal tract, immunosuppression (in the bone marrow), and hair loss (Carey and Burish 1988; McQuade et al. 2014). Biosurfactant compounds such as sophorolipids are naturally produced agents that may possess the ability to differentially affect cancer and normal epithelial cells and as such their anti-cancer activities are a growing area of research. Here, we investigated the in vitro effects of a 94% pure preparation of ASL on a non-transformed intestinal epithelial cell line (CCD-841-CoN) and 4 colorectal cancer cell lines (HT29, HT115, Caco2, and HCT116). Colonic epithelia (CCD-841-CoN) treated with ASL showed no sign of toxicity as evidenced by maintenance of their viability at doses ranging between 0.001 and 100 µg mL^−1^. However, at doses of 20 µg mL^−1^ and above, ASLs potently reduced cell viability in all the colorectal cancer cell lines examined. Although the cytotoxic potency of sophorolipid mixtures has been reported against cancer cell lines previously (Chen et al. 2006b; Fu et al. 2008; Shao et al. 2012; Ribeiro et al. 2015; Callaghan et al. 2016; Adu et al. 2022), this is the first time an anti-cancer effect has been reported from a purified and well characterised ASL on a range of cell lines from the same tissue of origin (i.e. colorectal cancer cells).

Importantly, the ASL preparation utilised here exhibited a differential effect in vitro on non-transformed cell lines compared to CRC cells, with a dose-dependent cytotoxic response in all five CRC cell lines. A differential effect has been reported in the previous studies where SLs were cytotoxic to pancreatic, liver, and melanoma cancer cell lines but with no demonstrated toxicity to non-transformed cell lines (Fu et al. 2008; Adu et al. 2022). However, Fu et al. (2008) utilised non-adherent circulating blood monocytes (PBMC) as a control for adherent transformed cells making conclusions from this study difficult to interpret. Therefore, other than Adu and colleagues work on melanoma cell lines, a directly comparable description of the specificity of a pure preparation of ASL that uses a normal adherent cell line from the same tissue of origin has not been carried out.

It has been hypothesised that SL can intercalate into the cytoskeleton of cells resulting in membrane disruption. Changes in cell morphology consistent with disruption of membrane cytoskeletal protein distribution have previously been shown to occur with SL doses as low as 30 µg mL^−1^ in pancreatic H7402 and lung cancer A549 cell lines (Chen et al. 2006b). Our study demonstrated that a preparation dominated by a single congener of ASL at low concentrations (40 µg mL^−1^) induced cell rounding, cytoplasmic condensation and cell detachment in all the CRC cancer cell lines tested, with the adenocarcinoma cell lines HT29 and Caco2 being most susceptible. At doses of 70–100 µg mL^−1^, ASL caused a significant increase in the numbers of detached cells with all cells in the supernatant from treated cultures showing morphological features consistent with apoptosis or necrosis. Similar results have been observed in HepG2 liver cancer cells treated with LSL preparations (Wang et al. 2021).

ASLs, used at the doses described in our study, induced a reduction of tension at the interfacial region of the bilayer resulting in phospholipid dehydration which affected lipid stability and ultimately resulted in cell death (Maget-Dana et al. 1989; Shah et al. 2005; Ortiz et al. 2009). To assess cell death mechanisms caused by ASLs, ethidium bromide/acridine orange staining was carried out allowing the morphological identification of the type of cell death. We have conclusively demonstrated that ASL induces both apoptosis and necrosis in these cancer cell lines in a dose-dependent manner. The ability of ASL to induce either apoptosis or necrosis may be cell-line specific, as high proportions of apoptotic cells have been reported following the addition of diacetylated LSL to liver (H7402) and lung cancer (H7402) cell cultures, while necrosis is primarily observed in a pancreatic carcinoma cell line (HPAC) treated with sophorolipid mixtures, LSL, or methyl ester derivative SL (Shao et al. 2012).

An important characteristic of malignant growth is the ability of tumour cells to leave their restricted compartment and gain access to blood vessels to initiate the first phase of metastasis (Hanahan and Weinberg 2011). The movement of cells across tissues therefore plays an important role in this progression thus highlighting the need for an agent that can counteract the migratory and diapedesis properties of colorectal cancer cells (Dianzani et al. 2014). It has been postulated that the amphiphilic properties of sophorolipids permit their incorporation into the mammalian cellular membrane disrupting cellular functions such as proliferation and migration (Zhao et al. 2013; Haque et al. 2021; Adu et al. 2022). We assessed the ability of ASLs to inhibit migration of the CRC cell lines HT29 and HT115. At a low dose (10 µg mL^−1^) of ASL, there was no effect on normal colonic epithelial cells (CCD-841-CoN) and scratch coverage after 72 h. However, the same dose of ASL applied to colorectal cancer cell lines HT29 and HT115 cultures reduced the total percentage area of scratch covered to between 12 and 25%. An inhibition of migration induced by sophorolipid (as measured in the scratch assay) has been documented. In 2015, Riberiro and co-workers showed that 5 µg mL^−1^ of a 93% pure, diacetylated LSL resulted in significantly reduced migration of MDA-MB-231 breast cancer cells (Ribeiro et al. 2015), although to our knowledge, no reports are available for a study that solely utilises purified ASL. Adu et al. (2022) showed that the migration of a melanoma cell line SK-MEL-28 was significantly reduced when treated with LSL and ASL preparations in comparison with cells treated with a vehicle-only control, and immortalised keratinocytes (HaCaT) treated with the same sophorolipid preparations (Adu et al. 2022). The use of both migration and invasion assays incorporating the Boyden chamber is widely used to test candidate chemotherapeutics with the HT29 and HT115 cell lines commonly employed to test anti-invasive properties of potential chemotherapeutics in vitro (Li and Zhu 1999). Currently, the only studies investigating the potential anti-metastatic properties of biosurfactants are limited to the inhibitory effects on breast cancer cell lines MCF and MDA-MB-231 in vitro invasion mediated by the lipopeptide surfactin produced by *Bacillus* *subtilis* (Park et al. 2013). Addition of 10 µM (equivalent to approx. 660 µg mL^−1^ of ASL in this study) of surfactin had the ability to reduce the migration of MCF-7 and MDA-MB-231 through an extracellular matrix by 68% and 84% respectively (Park et al. 2013). The surfactin used by Park et al. (2013) also reduced the colony forming ability of both cell lines by 70% and 61%. To investigate the migration inhibitory properties of ASL, HT29 and HT115 cells were plated on a porous membrane and allowed to migrate through a septum in response to a FBS stimulus in the chamber below. ASL had no effect on the migration of normal colonic CCD-841-CoN cells; however, following treatment with 10 µg mL^−1^ and 50 µg mL^−1^ ASL, migration was significantly decreased in HT29 cell lines by 65% and 72% respectively, and 86% and 81% in HT115 cell lines.

The anti-tumour activities of sophorolipid preparations in vivo are unclear. In a study by Li and co-workers, an intragastric administered mixture composed of 6 di-acetylated LSL congeners was shown to reduce the size of tumours in a murine HeLa xenograft model (Li et al. 2017). However, a purified form of LSL exaggerated the growth of neoplasm along the intestinal tract and increased intestinal blood loss in the Apc^min+/−^ mouse (Callaghan et al. 2016). In this study, we use the Apc^min+/−^ mouse model of FAP, to evaluate the chemotherapeutic potential of purified ASL in the treatment of solid neoplasms. Apc^min+/−^ mice develop intestinal adenomatous neoplasms (polyps), and animals typically present with enlarged spleens and reduced haematocrit by 4 months of age (Yekkala and Baudino 2007). This is an acute model with a life span of < 150 days, where the primary cause of death is not directly attributable to the development of numerous polyps but rather as a result of extensive intestinal bleeding and anaemia (Hinoi et al. 2007). When administered orally, topically, or via *i.v.* injection, sophorolipid mixtures are well tolerated (Ikeda et al. 1986). Similarly, in our study, both *wt* and Apc^min+/−^ mice tolerated the oral administration of ASL with no measurable gross anatomical or behavioural differences noted. Post-mortem analysis also revealed no effects on gross measures of peritoneal organs (size and dimensions) after ASL treatment. However, oral feeding of ASL to Apc^min+/−^ for 70 days resulted in a reduction of spleen size and a significant increase in haematocrit, consistent with decreased intestinal bleeding and improvement in the associated anaemia characteristic of this model (Perkins et al. 2002; Hinoi et al. 2007). This is a potentially significant finding, as rectal bleeding and anaemia are reported in over 30% of CRC patients and it is a contributing factor in reduced lifespan in both humans and Apc^min+/−^ mice (Ronnekleiv-Kelly and Kennedy 2011). In humans, laser ablation encourages coagulation of tumours demonstrating significant blood loss and this technique has shown to be effective after 2–5 treatments with a success rate of 80% (Kimmey 2004). However, recurrent bleeding episodes result in surgical intervention in 2–15% of patients (Rao et al. 2005). The oral administration of a well-tolerated non-toxic, pro-thrombotic agents to reduce intestinal blood loss in patients with haemorrhagic colorectal tumours may be a useful addition to the therapeutic treatment of these conditions.

In conclusion, the purified ASL mixture we utilised in this study differentially affects non-transformed in comparison to colorectal cancer cell lines, resulting in a significant and dose-dependent decrease in their viability, migration, and anchorage-independent growth characteristics. While ASL does not change either the size or number of intestinal polyps in Apc^min+/−^ mice, both spleen size and tumour-associated bleeding were reduced. This warrants further investigation of this orally available biosurfactant as a chemotherapeutic for delaying disease progression in pre-cancerous colorectal neoplasms.

## Supplementary Information

Below is the link to the electronic supplementary material.

Supplementary file1 (PDF 482 KB)

## Data Availability

The datasets generated and/or analysed during the current study are available from the corresponding authors on reasonable request.
