# Supplementary material for: Microbial sophorolipids inhibit colorectal tumour cell growth in vitro and restore haematocrit in Apcmin+/− mice
Source: Appl Microbiol Biotechnol. 2022 Aug 15;106(18):6003–16. doi: 10.1007/s00253-022-12115-6 (PMC9467956; doi:10.1007/s00253-022-12115-6)
Supplement: Supplementary file 1 — Supplementary file1 (PDF 482 KB) [file 253_2022_12115_MOESM1_ESM.pdf]

APPLIED MICROBIOLOGY AND BIOTECHNOLOGY

Supplementary material for:

Microbial sophorolipids inhibit colorectal tumour cell growth *in vitro* and restore haematocrit in Apc$^{min+/-}$ mice

Breedge Callaghan[1], Matthew S. Twigg[1], Niki Baccile[2], Inge N. A. Van Bogaert[3], Roger Marchant[1], Christopher A. Mitchell[1,*] & Ibrahim M. Banat[1,*]

*Corresponding Authors – Ibrahim Banat and Christopher Mitchell

Email: im.banat@ulster.ac.uk, ca.mitchell@ulster.ac.uk

Tel: +44 28 7012 3062, +44 28 7012 4089

1. School of Biomedical Sciences, Ulster University, Coleraine, BT52 1SA, U.K.
2. Sorbonne Universités, UPMC Univ Paris 06, CNRS, Collège de France UMR 7574, Chimie de la Matière Condensée de Paris, UMR 7574, 75005 Paris, France.
3. Centre for Synthetic Biology, Department of Biotechnology, Ghent University, Coupure Links 653, 9000 Ghent, Belgium.

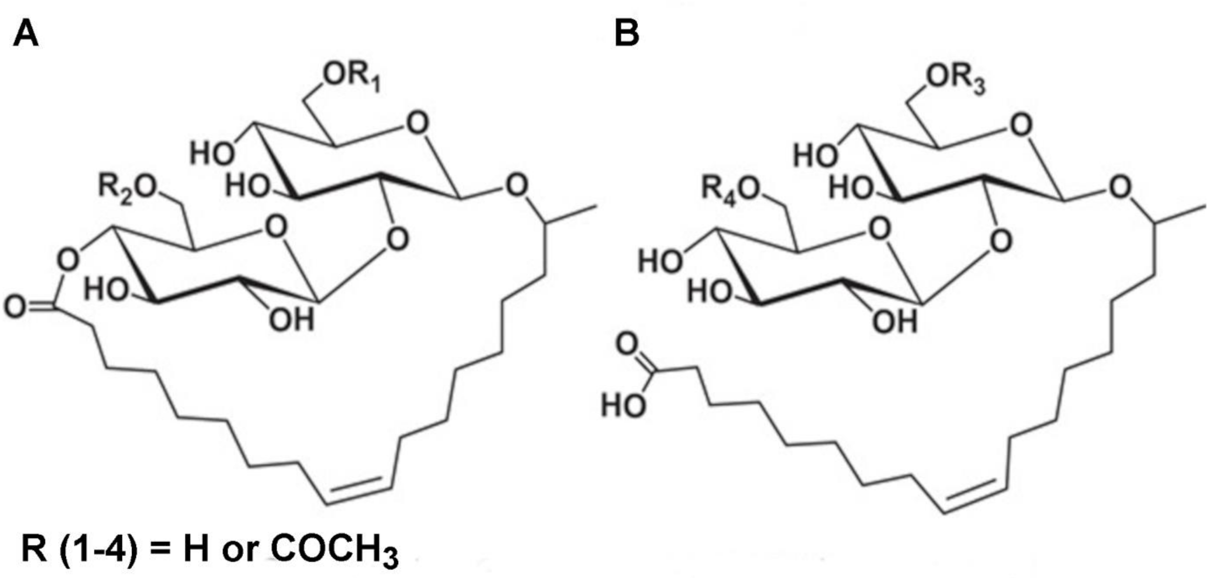

Supplementary Figure 1

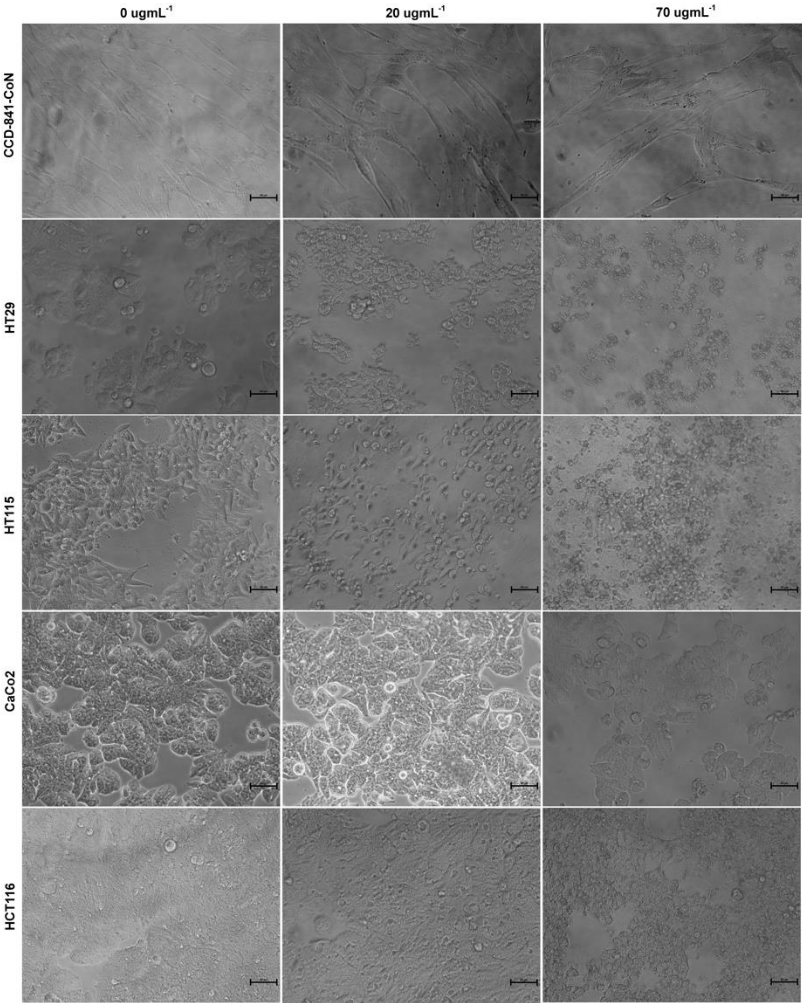

Supplementary Figure 2

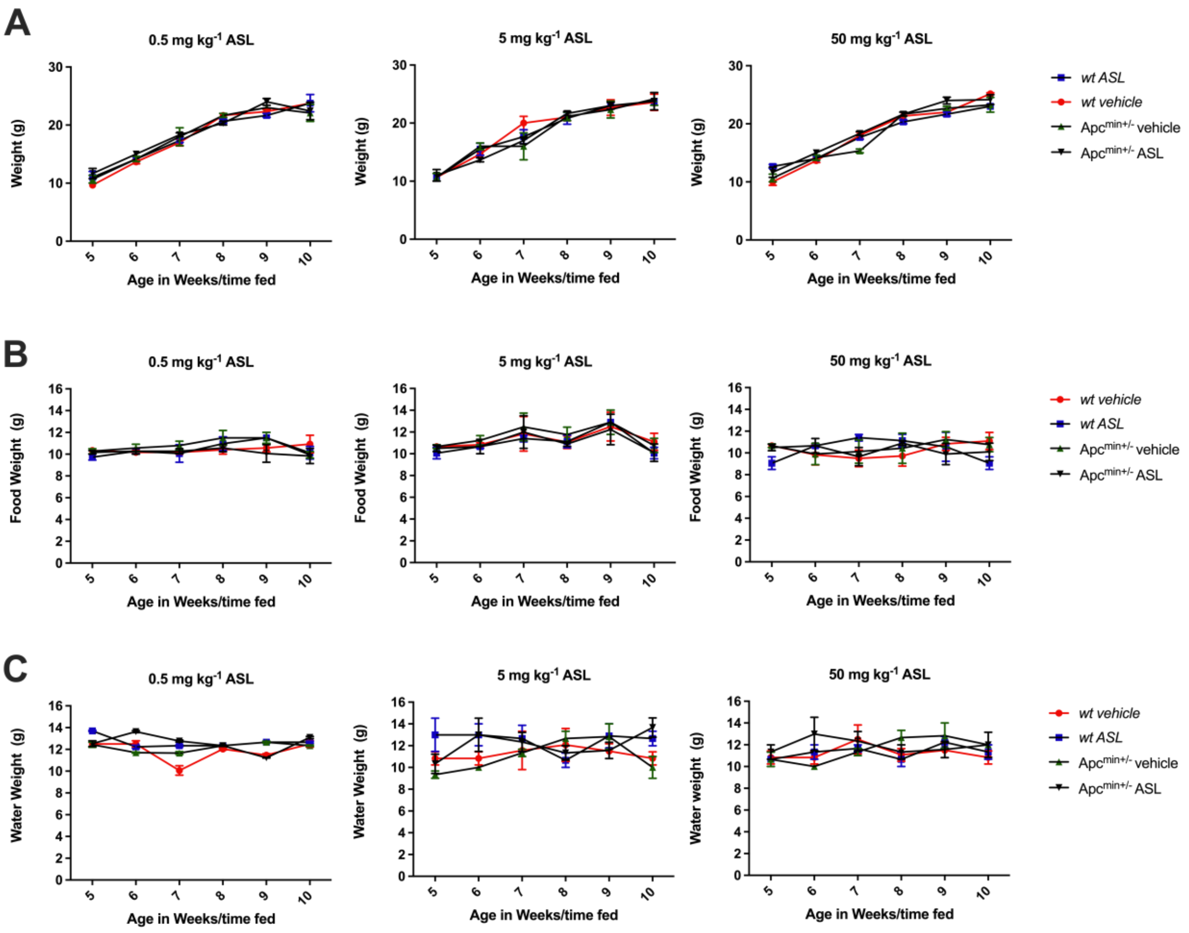

Supplementary Figure 3

Supplementary Figure Legends

Supplementary Figure 1. Generalized molecular lactonic sophorolipids (LSL's) (A) and acidic sophorolipids (ASL's) (B). Fatty acid chains shown here represent the most common C18 sophorolipid congeners.

Supplementary Figure 2. Randomly selected imaged of all four colorectal cancer cells lines treated for 24 h with either 40 or 70 μg mL^{-1} ASL and vehicle-only control (0 μg mL^{-1}). Changes in cell morphology were observed in all colorectal cancer cells lines treated with 70 μg mL^{-1} ASL, while CCD-841-CoN cell treated under the same conditions retained normal cellular morphology.

Supplementary Figure 3. APC$^{min+/-}$ and *wt* mice were fed vehicle-only or 0.5, 5 or 50 mg kg^{-1} ASL every other day for 5 weeks and monitored closely. Weight gain (**A**), food consumption (**B**) and water consumption (**C**) were

measured on a weekly basis. There were no significant differences in these three parameters between each cohort of mice. Values are represented by mean, n = 3 per cohort.

Supplementary table 1

**Retention Time (min)**	**ASL Congener***	**MW**	**% Relative Abundance**
12.02	C16:1s	593	1.0
12.16	C16:0s	595	2.0
12.37	C16:0t	595	0.0
12.40	C18:2s	619	1.0
12.54	C18:2t	619	10.0
12.87	C18:1s	622	75.0
13.27	C18:1t	622	10.0
13.64	C18:0s	624	1.0

* s = sub-terminal hydroxylation site, t = terminal hydroxylation site

Supplementary table 2

	wt **vehicle-only**	***wt*** **ASL**	**APC$^{min+/-}$ vehicle-only**	**APC$^{min+/-}$ ASL**
**Liver**	0.740 g (± 0.04)	0.700 g (± 0.03)	0.750 g (± 0.01)	0.729 g (± 0.02)
**Stomach**	0.525 g (± 0.01)	0.551 g (± 0.02)	0.550 g (± 0.03)	0.520 g (± 0.01)
**Kidneys**	0.420 g (± 0.02)	0.401 g (± 0.02)	0.440 g (± 0.01)	0.413 g (± 0.06)
**Lungs**	0.110 g (± 0.03)	0.100 g (± 0.01)	0.140 g (± 0.01)	0.120 g (± 0.01)
**Heart**	0.140 g (± 0.05)	0.120 g (± 0.07)	0.114 g (± 0.04)	0.100 g (± 0.03)
**Spleen**	0.180 g (± 0.02)	0.160 g (± 0.02)	0.562 g (± 0.05)	0.560 g (± 0.07)
**Pancreas**	0.179 g (± 0.04)	0.189 g (± 0.03)	0.190 g (± 0.03)	0.180 g (± 0.05)

Supplementary Table Headings

Supplementary table 1. Table showing the percentage abundance of each nonacetylated acidic SL congener present in the sample that was utilized for all *in vitro* and *in vivo* work presented in this study. The data was collated on the basis of the HPLC-ELSD integrated area values. The most abundant congener in the sample was found to be acidic-SL C18:1s.

Supplementary table 2. Mean weights of organs dissected from mice from each treatment cohort fed with 50 mg kg^{-1} ASL. No significant difference in weight was observed in any organ between mice fed with vehicle only control or ASL.
